# Evaluation of TLSOS in the bracing in adolescent idiopathic scoliosis trial (BrAIST)

**DOI:** 10.1186/1748-7161-8-S1-O48

**Published:** 2013-06-03

**Authors:** LA Dolan, KA Haggerty, SL Weinstein

**Affiliations:** 1 University of Iowa Department of Orthopaedics and Rehabilitation, Iowa City, IA, USA

## Background

The success of TLSOs in reducing the risk of curve progression varies widely across published reports. This variation has been linked to patient risk factors such as initial Cobb angle and maturity, and time in brace. Percentage correction has also been associated with outcomes. [1-3] However, no reports have examined characteristics of individual braces, and how these characteristics influence percentage correction, compliance, and eventual outcome.

## Aims

To report the BrAIST Bracing Evaluation Committee findings to-date.

## Methods

BrAIST includes evaluation of the bracing customization process and immediate outcome of each brace worn in the trial. Each is reviewed by an independent committee of at least 2 orthotists, and 2 pediatric orthopedic surgeons. The committee reviews all orthotist reports and pertinent radiographs (in-brace, initial out-of-brace and side-bending films). Customization process indicators include brace trimlines, pad placement, total contact fit, and upper thoracic alignment; outcome indicators include curve correction relative to flexibility and decompensation. Overall process and outcome are classified as either "satisfactory" or "unsatisfactory" based on committee consensus. In case of a tie, the classification is based on the orthotists' evaluation.

## Results

76 braces were reviewed from 20 institutions. In general, the orthotists responded with more detailed and critical comments than the surgeons. 62% of brace had a "satisfactory" customization process, and 63% a "satisfactory" immediate outcome. The average correction was 33%; only 25% of the braces resulted in >50% correction. Satisfactory process was associated with greater in-brace curve correction. In all but 7%, both the process and outcome carried the same evaluation. Process and outcome varied greatly across institutions, but the number of reviews is too small at this time to draw any conclusions.

## Conclusions

Only 40% of the braces have been reviewed, but there is a clear trend linking proper customization process with the immediate outcome. The majority of braces did not achieve the goal of 50% correction suggested by the literature. Variation between centers indicates that not all orthotists are equally skilled at brace customization.
